# Intracystic papillary neoplasm of the gallbladder concomitant with xanthogranulomatous cholecystitis: a case report

**DOI:** 10.1186/s40792-021-01312-6

**Published:** 2021-10-24

**Authors:** Takashi Aida, Masashi Tsunematsu, Kenei Furukawa, Koichiro Haruki, Yoshihiro Shirai, Shinji Onda, Yoichi Toyama, Kazutaka Gomisawa, Hiroyuki Takahashi, Toru Ikegami

**Affiliations:** 1 Division of Hepatobiliary and Pancreatic Surgery, Department of Surgery, The Jikei University School of Medicine, 3-25-8, Nishi-Shinbashi, Minato-ku, Tokyo, 105-8461 Japan; 2 Department of Pathology, The Jikei University School of Medicine, Tokyo, Japan

**Keywords:** Intracystic papillary neoplasm, Gallbladder, Xanthogranulomatous cholecystitis

## Abstract

**Background:**

The intracystic papillary neoplasm (ICPN) is a newly established disease concept. It has been regarded as a preinvasive neoplastic lesion, similar to intraductal papillary mucinous neoplasm of the pancreas. Limited information is available on the clinical and imaging features of ICPN.

**Case presentation:**

A 65-year-old woman was referred to our hospital for assessment of a gallbladder tumor. Contrast-enhanced computed tomography showed a papillary tumor in the fundus of the gallbladder with irregular thickening of the gallbladder wall that spread into the cystic duct. The boundary between the tumor and liver was unclear. The patient was diagnosed with gallbladder cancer with liver invasion. We performed extended cholecystectomy with liver bed resection after confirming the absence of cancer cells in the resection margin of the cystic duct. After pathological examination, the tumor was diagnosed as an ICPN with xanthogranulomatous cholecystitis. The patient was discharged on postoperative day 8 with no complications.

**Conclusions:**

We have described a rare case of ICPN concomitant with xanthogranulomatous cholecystitis. Clinicians should include ICPN as a differential diagnosis in patients with a papillary or polypoid tumor in the gallbladder.

## Background

An intracystic papillary neoplasm (ICPN) is classified as a premalignant gallbladder lesion in the 2018 classification of the World Health Organization [1]. However, the morphological characteristics of ICPN remain unclear because of its rarity. Additionally, it is sometimes difficult to discriminate gallbladder carcinomas from cholecystitis during the preoperative period [2].

We herein report a case of ICPN concomitant with xanthogranulomatous cholecystitis (XGC) and review previously published reports of ICPN.

## Case presentation

**Fig. 1:**
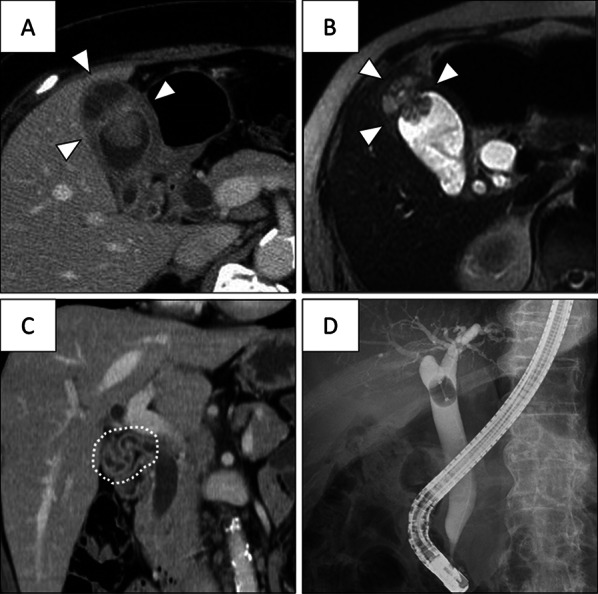
Enhanced computed tomography and T2-weighted magnetic resonance imaging. **A**, **B** A papillary or polypoid tumor was present in the gallbladder. **C** The gallbladder wall was generally thickened, and the thickening continued to the cystic duct. **D** Endoscopic retrograde cholangiography revealed a filling defect in the cystic duct

A 65-year-old woman was referred to our hospital for assessment of a gallbladder tumor that had been detected by abdominal ultrasonography during a medical checkup. She had no symptoms. Contrast-enhanced computed tomography revealed a papillary lesion (25 mm in diameter) in the fundus of the gallbladder with irregular thickening of the gallbladder wall. The boundary between the tumor and liver was unclear, and the wall thickening extended from the fundus to the cystic duct (Fig. 1A–C). Endoscopic retrograde cholangiography showed a complete filling defect in the cystic duct (Fig. 1D). Blood test showed inflammatory maker did not elevate. Serum carcinoembryonic antigen level was 2.1 ng/ml, and serum carbohydrate antigen 19-9 level was 24 U/ml. According to these findings, we diagnosed the tumor as gallbladder cancer invading the liver and cystic duct, and we decided to perform extended cholecystectomy.

There were no adhesions around the gallbladder, while the gallbladder wall was thickened. The hard tumor was detected at the fundus. Intraoperative ultrasonography revealed the tumor did not invade liver obviously. Inflammation changes were seen around the neck and Calot triangle. After ligation and dissection of the cystic duct at the junction, the intraoperative frozen section of the cystic duct stump and 2 sentinel lymph nodes were negative for malignancy; therefore, we performed extended cholecystectomy with liver bed resection.

**Fig. 2:**
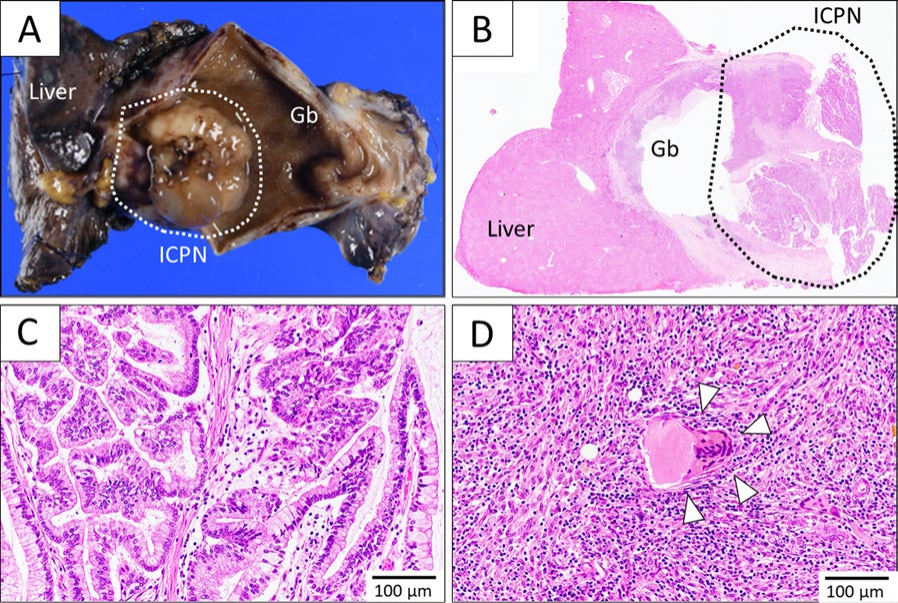
Pathological findings. **A** Gross inspection of the resected specimens revealed that the mass had extroversive development from the mucosa of the gallbladder and contained some mucinous cysts. The mucus adhered to the surface of tumors. **B–D** Hematoxylin and eosin-stained histological sections. **B** Magnification × 40. **C** Magnification × 200. **D** Magnification × 200. The papillary growth was composed of atypical cells along with the development of fibrovascular stalks. Some of these tall columnar cells contained rich mucus. There was no evidence of tumor invasion. The gallbladder wall was highly thickened by the presence of severe inflammatory lesions containing lymphocytes and multinucleated giant cells, which were considered to indicate chronic granulomatous changes in the wall. The histological diagnosis was intracystic papillary neoplasm with xanthogranulomatous cholecystitis

Macroscopic examination of the resected specimen showed a superficially spreading papillary tumor with thick mucus on its surface (Fig. 2A, B). The gallbladder wall was diffusely thickened. Pathological examination revealed that the gallbladder neoplasm was composed of atypical cells arranged in a papillary architecture along with the development of fibrovascular stalks. These tall columnar cells contained large amounts of mucus (Fig. 2C). Ovarian-like stroma was not detected. The nucleolus body was remarkable, but the tumor was noninvasive and showed no evidence of lymph node metastasis. These pathological findings were compatible with ICPN. In immunohistochemical staining, MUC5AC and MUC6 were strongly positive. CK7 and MUC1 were also positive, but not CK20, MUC2, estrogen receptor, and progesterone receptor. Immunohistochemistry indicated that ICPN was predominantly gastric type, with focal pancreatobiliary type. In addition, many lymphocytes and multinucleated giant cells had infiltrated the thickened gallbladder wall with prominent Rokitansky–Aschoff sinuses. These finding were especially seen at the fundus and were indicative of chronic granulomatous changes within the fundus (Fig. 2D). Based on these pathological findings, we diagnosed the tumor as an ICPN concomitant with XGC.

The patient was discharged on postoperative day 8 with no complications. She was clinically well with no evidence of recurrence at 3 months after resection.

## Discussion

The ICPN is a relatively new disease concept that was first described in the 2010 World Health Organization classification [3]. It has been regarded as a counterpart disease of intraductal papillary neoplasm of the bile duct and intraductal papillary mucinous neoplasm of the pancreas. ICPNs more often occur in women older than 60 years, and their incidence in women is twice as high as that in men [1]. Almost 50% of patients with ICPNs develop abdominal pain in the peripheral aspect of the upper quadrant, while the remaining 50% are asymptomatic and incidentally found to have a tumor, as in our case [4]. ICPNs are considered to be precancerous lesions. The prognosis of ICPNs is much better than that of invasive gallbladder carcinomas. In fact, the 3-year survival rate of patients with noninvasive and invasive ICPN is 90% and 60%, respectively [4].

The pathological characteristics of ICPN are macroscopic papillary growth within the gallbladder, regardless of mucin production, and the microscopic presence of intraductal papillary growth with delicate fibrovascular stalks [5]. Therefore, ICPNs can be diagnosed only after analysis of resected specimens, and they are found in < 0.5% of cholecystectomies [4, 6]. Because of the limited number of case reports, the main characteristics of ICPNs remain unclear. We searched PubMed using the keywords “gallbladder” and “intracystic papillary neoplasm” or “intracholecystic papillary neoplasm” from 2010 to 2021 and found 11 case reports of ICPNs [5, 7–14]. We summarized 12 cases (all 11 previously published cases in addition to the present case) in Table 1, focusing on the imaging features and preoperative diagnoses.

**Table 1:** Clinical characteristics of patients with ICPN

Case	Age	Sex	Symptoms	Imaging	Diagnosis	Surgery	Outcome	References
1	48	F	No	Cystic tumor	–	LC	n.d	[7]
2	71	F	Epigastric pain	Wall thickening	–	LC	Alive 30 months	[8]
3	86	F	Jaundice	Papillary tumor, wall thickening	ICPN	LC	n.d	[9]
4	58	F	Fever	Papillary tumor	ICPN	SSPPD	Alive 6 months	[10]
5	71	M	No	Papillary tumor	ICPN	LC	n.d	[11]
6	78	F	Epigastric pain	Papillary tumor	Gallbladder tumor	LC	Alive 12 months	[5]
7	64	M	Epigastric pain	Cystic tumor	Gallbladder tumor	LC	n.d	[12]
8	54	F	Epigastric pain	Papillary and nodular tumor	Gallbladder tumor	ExC	Alive 2 months	[13]
9	74	F	No	Papillary tumor	GbC	ExC	n.d	[14]
10	61	F	No	Papillary tumor	GbC	ExC	n.d	[14]
11	83	M	No	Papillary tumor	GbC	ExC	n.d	[14]
12	65	F	No	Papillary tumor, Wall thickening	GbC	ExC	Alive 2 months	Present case

*ExC* extended cholecystectomy, *F* female, *GbC* gallbladder cancer, *ICPN* intracystic papillary neoplasm, *LC* laparoscopic cholecystectomy, *M* male, *n.d* no data, *SSPPD* subtotal stomach preserving pancreaticoduodenectomy

Of these 12 cases, papillary or polypoid lesions were present in the gallbladder in 9 cases (75%). However, gallbladder wall thickening was uncommon. ICPN could be diagnosed preoperatively in only three cases. Two patients underwent biopsy using peroral cholangioscopy, and another patient underwent cytology using endoscopic naso-gallbladder drainage. These results suggest that biopsy may help to achieve a definitive diagnosis in patients with papillary or polypoid lesions. One patient with a preoperative diagnosis of ICPN located only in the gallbladder underwent laparoscopic cholecystectomy without extended resection.

In our case, because computed tomography and magnetic resonance imaging showed not only a papillary tumor, but also irregular wall thickening, the possibility of gallbladder carcinoma needed to be considered. The radiological findings of XGC, such as the various patterns of wall thickening, resemble those of gallbladder carcinomas [15]. Therefore, the presence of XGC made a precise diagnosis difficult to achieve in the current case. Moreover, the mucus produced by the ICPN obstructed the thickened and narrowed gallbladder duct, preventing cytology and biopsy via the duct during endoscopic retrograde cholangiography. Given that intraoperative frozen section analysis is useful in distinguishing XGC and gallbladder carcinomas [16], careful intraoperative diagnosis may help us to choose the optimal operative procedure.

## Conclusions

We have herein reported a rare case of ICPN with XGC. Clinicians should include ICPN as a differential diagnosis in patients with a papillary or polypoid tumor in the gallbladder, keeping in mind that biopsy may allow for a definitive diagnosis.

## Data Availability

The data that support the findings of this study are available from the corresponding author upon reasonable request.

## Abbreviations

ICPN: Intracystic papillary neoplasm
XGC: Xanthogranulomatous cholecystitis
